# Targeted expansion of a barley genebank core collection facilitates the discovery of disease resistance loci

**DOI:** 10.1007/s00122-025-05139-9

**Published:** 2026-01-11

**Authors:** Zhihui Yuan, Yusheng Zhao, Klaus Oldach, Ahmed Jahoor, Jens Due Jensen, Viktoria-Elisabeth Dohrendorf, Tobias W. Eschholz, Sabrina Roescher, Nils Stein, Jochen C. Reif, Samira El Hanafi

**Affiliations:** 1 Leibniz Institute of Plant Genetics and Crop Plant Research (IPK) Gatersleben, Seeland, Germany; 2 KWS LOCHOW GmbH, Ferdinand-Von-Lochow-Str. 5, 29303 Bergen, Germany; 3 Nordic Seed Germany GmbH, Kirchhorster Str. 16, 31688 Nienstädt, Germany; 4 Nordsaat Saatzucht GmbH, Zuchtstation Gudow, Hofweg 8, 23899 Gudow, Germany; 5 Limagrain GmbH, Salderstr. 4, 31226 Peine-Rosenthal, Germany; 6 Crop Plant Genetics, Institute of Agricultural and Nutritional Sciences, Martin-Luther-University of Halle-Wittenberg, Halle (Saale), Germany

## Abstract

Utilizing the diversity preserved in genebank collections is essential for accelerating crop improvement, yet information is often limited to selected core collections. Genome-wide prediction (GWP) offers a promising approach to large-scale phenotypic imputation, with proven utility in practical pre-breeding contexts. In this study, we leveraged GWP to expand the German Federal ex situ barley core collection (core1000) with a focus on resistance to *Puccinia hordei*, *Blumeria graminis hordei*, and *Rhynchosporium commune*. Using the barley core1000 collection, which was originally selected to maximize molecular diversity, we trained genomic prediction models and imputed resistance scores for 20,458 genebank accessions based on sequence data encompassing 306,049 high-quality SNPs. To empirically validate prediction accuracy, we selected 300 spring and winter barley genotypes for field evaluation across four environments, resulting in moderate-to-strong correlations between predicted and observed resistance levels. Genome-wide association mapping in this set revealed five marker–trait associations that were not detected in the original core1000 collection. These results demonstrate that prediction-informed sampling can effectively expand trait-relevant genetic diversity and increase the frequency of resistance-associated alleles, thereby improving the power to detect loci that may be overlooked in conventional panels. Accordingly, GWP supports the targeted inclusion of accessions with trait-relevant variation and enhances the value of genebank resources for trait discovery and pre-breeding applications.

**Supplementary Information:**

The online version contains supplementary material available at 10.1007/s00122-025-05139-9.

## Introduction

Plant genetic resources preserved in ex situ genebanks are invaluable for enhancing crop resilience to biotic and abiotic stresses. However, their use in pre-breeding programs is limited. This is largely due to the insufficient phenotypic information available for thousands of accessions (Keilwagen et al. 2014). Since fully characterizing entire genebank collections is resource-intensive and time-consuming, efforts have increasingly focused on scalable alternatives. Core collections are therefore assembled from large ex situ genebank holdings to capture the maximum possible molecular diversity in a reduced number of accessions. This enables systematic phenotyping and deep sequencing for efficient downstream usage. However, the resulting phenotypic distribution may not reflect the full spectrum of relevant traits. The distribution may be skewed toward the undesired fraction, due to the prioritization of molecular diversity over trait-specific variation. For example, the barley (*Hordeum vulgare* L.) core1000 collection, generated from a global panel of 22,626 accessions maintained at the Federal ex situ Genebank for Agricultural and Horticultural Crops at IPK Gatersleben (Milner et al. 2019), showed a bias toward susceptibility to diseases (Yuan et al. 2024b). Such imbalances may omit rare beneficial alleles. Overcoming this constraint requires a targeted, trait-specific expansion without phenotyping the entire genebank. Addressing this challenge calls for predictive strategies that can guide the selection beyond the current core collection.

Genome-wide prediction (GWP) is a promising solution to impute trait performance using genomic data, trained on a genetically diverse and phenotyped core collection (Jiang et al. 2021; Schulthess et al. 2022; Berkner et al. 2022; El Hanafi et al. 2023). This strategy has been applied to predict agronomic traits in non-phenotyped accessions on a large scale (Crossa et al. 2016; Yu et al. 2016; Thorwarth et al. 2018; Zhao et al. 2021; Gonzalez et al. 2021). However, predictions in genebank contexts are often not validated beyond classical cross-validation schemes. This leaves an uncertainty, particularly for complex, environment-sensitive traits like disease resistance (Yu et al. 2016; Tiezzi et al. 2017; Toghiani et al. 2024), which demands for independent validation. In parallel, genome-wide association studies (GWAS) provide complementary opportunities to identify trait-associated loci that go beyond purely predictive modeling. Together, predictive approaches coupled with robust validation and GWAS-guided discovery can enhance the molecular diversity captured in genebank panels, increasing their value for pre-breeding.

Our study implements this validation framework using a previously published genomic dataset comprising 306,049 high-quality single-nucleotide polymorphisms (SNPs) for 20,458 barley accessions (Milner et al. 2019), along with phenotypic data on resistance against *Puccinia hordei* (LR), *Blumeria graminis hordei* (PM), and *Rhynchosporium commune* (RHY; Yuan et al. 2025) of the core1000 collection used as a training set. By integrating genomic prediction, empirical field validation, and genome-wide association mapping, we evaluated the effectiveness of a prediction-informed strategy in enriching resistance-relevant diversity for core collections and facilitating the discovery of associated loci. In particular, our objectives were to (1) assess the cross-validated prediction accuracy within the barley genebank core1000 collection, (2) predict the resistance performance of all non-phenotyped genebank accessions, (3) select 300 genotypes as a validation set for further empirical experiments across four environments, and (4) identify trait-associated loci based on GWAS conducted in the validation panel.

## Materials and methods

### Phenotypic and genomic data records

This study builds on previously published genomic information (Milner et al. 2019) of 22,626 barley accessions of the IPK genebank resulting in 306,049 genotyping-by-sequencing (GBS)-based SNPs. The genomic data were combined with the phenotypic data that include disease scores for powdery mildew (PM; caused by *Blumeria graminis hordei*), leaf rust (LR; caused by *Puccinia hordei*), and scald (RHY; caused by *Rhynchosporium commune*) from a barley genebank core1000 collection (565 spring barley and 288 winter barley). Phenotyping was conducted over three years (2020, 2021, and 2022) across eight environments throughout Germany (Yuan et al. 2025). Disease severity was assessed under natural field infection and scored using an ordinal scale from 1 (fully resistant) to 9 (fully susceptible) following the guidelines of the German Federal Plant Variety Office (Bundessortenamt 2000). Linear mixed models were implemented in conjunction with routines for data quality assessment, modeling the phenotypes as a function of the genotype, year, and residual variance. The quality and reliability of the phenotypic data were rigorously assessed by estimating trait-specific heritability estimates, which, after outlier correction, were generally high and exceeded 0.77 across winter and spring, except for *Rhynchosporium commune* in spring population. Best linear unbiased estimations (BLUEs) of all phenotyped accessions were used for each trait as described in Yuan et al. (2025).

### Genomic prediction for disease traits

Using the aforementioned genomic information including 306,049 GBS-derived SNPs, genome-wide prediction of the three aforementioned disease traits was performed using the genome-wide best linear unbiased prediction model (GBLUP; VanRaden 2008). Briefly, the model is described as follows:

1 $$y = X\beta + g + e$$

where *y* is an n-dimensional vector of the observed phenotypic values (*n* is the number of genotypes), *β* is the common intercept, and *X* is a column vector of ones (*X* = 1_n_). The term *g* is the n-dimensional vector of the genetic values, and *e* is the residual term. In the model, *β* is treated as a fixed effect, whereas $g \sim N(0,{\sigma }_{\mathrm{g}}^{2}G)$ and $e\sim N(0,{\sigma }_{\mathrm{e}}^{2}I)$ were random effects, where *G* is the marker-derived additive genomic relationship matrix as in VanRaden (2008), *I* is the identity matrix, and ${\sigma }_{\mathrm{g}}^{2}$ and ${\sigma }_{\mathrm{e}}^{2}$ are the corresponding genetic and residual variance components, respectively. The mixed model equations for genome-wide prediction were implemented using the R package BGLR (Pérez and De Los Campos 2014), assuming an inverse *χ*^2^ prior distribution for ${\sigma }_{\mathrm{g}}^{2}$.

Fivefold cross-validation was applied separately within each growth class among the phenotyped genebank accessions (565 spring barley and 288 winter barley). For each growth class, accessions were randomly divided into five subsets, each with balanced proportions of accessions, of which four subsets served as the training set, with the remaining as the test set. The random sampling was repeated 100 times, resulting in 500 cross-validation runs. The prediction ability was estimated as the Pearson correlation coefficient of the predicted and observed values. The cross-validation was implemented in R package BWGS (v0.2.1; Charmet et al. 2020).

The phenotyped core1000 collection was used as a training set to predict the performance of non-phenotyped accessions within the genebank, separately for the spring and winter populations. The reliability of the prediction of each individual was assessed according to Mrode (2014) as follows (2):

2 $${\mathrm{reliability}} = 1 - \frac{{{\mathrm{PEV}}}}{{\sigma_{{\mathrm{g}}}^{2} }}$$

where PEV and ${\sigma }_{\mathrm{g}}^{2}$ are the estimated error and the additive genetic variances, respectively. This metric reflects the proportion of genetic variance captured by the prediction and provides an indication of confidence in the predicted performance.

### The selection and field test for the validation set

To validate our genomic predictions, we selected 300 genotypes (150 spring and 150 winter barley) from the test set, guided by the predicted phenotype values, prediction reliability estimates, and their seed availability (Supplementary Table S1) based on a trait-specific selection strategy. Selection followed a trait-specific strategy in which each of the three targeted traits the subpopulations were independently chosen based on their low predicted disease score (GEBV < 4) and high reliability estimates (> 0.5), with a few exceptions due to limited seed availability. These subsets were then combined to form the final validation set.

The selected populations were subsequently evaluated during the 2023–2024 cropping season following a generalized alpha lattice design with two-row plots as the experimental unit. Field trials were conducted in four environments in Germany (Supplementary Figure S1): Bergen (52°48′26.9"N, 9°59′55.5"E; 10.9 °C average annual temperature; 565.3 mm average annual rainfall); Nienstädt (52°17′35.52"N, 9°08′57.156"E; 10.7 °C average annual temperature; 638.4 mm average annual rainfall); Peine-Rosenthal (52°18′09.828"N, 10°10′28.488"E; 10.9 °C average annual temperature; 607.5 mm average annual rainfall); Gudow (53°33′28.0"N, 10°47′50.5"E; 10.4 °C average annual temperature; 581.1 mm average annual rainfall). All trials were unreplicated except at Nienstädt, where two replications were considered. Disease severity for powdery mildew, leaf rust, and scald was assessed under natural field infection, applying the same ordinal scale described previously (Bundessortenamt 2000).

### Phenotypic data analysis for the validation population

Statistical analysis was separately performed for spring and winter barley phenotypic data. Variance components and BLUEs were computed for each genotype using the following linear mixed model (LMM):

3 $$y_{ijkm} = \mu + E_{m} + g_{i} + g_{i} \times E_{m} + E_{m} :r_{j} :b_{k} + e_{ijkm} ,$$

where *y*_*ijkm*_ denoted the vector of phenotypic values for *i*^*th*^ genotype (*g*) tested in *k*^*th*^ block (*b*) nested in *j*^*th*^ replication (*r*) in *m*^*th*^ environment (*E*), *μ* was the common mean, and *e* denoted the error term of the model. We assumed that all random effects followed an independent normal distribution with different variance components.

Due to the model convergence constraints, for RHY and LR scored in the spring population, the previously described model (3) was reduced to:

4 $$y_{ikm} = \mu + E_{m} + g_{i} + e_{ikm} ,$$

In models (3) and (4), all terms except *µ* and *g*_*i*_ were considered random when deriving BLUEs across environments, whereas only *µ* was treated as fixed to estimate variance components and hence to compute heritability following the model:

5 $$h^{2} = \frac{{\sigma_{{\mathrm{g}}}^{2} }}{{\sigma_{{\mathrm{g}}}^{2} + \frac{{\sigma_{{{\mathrm{g}} \times {\mathrm{E}}}}^{2} }}{{\overline{n}_{{\mathrm{E}}} }} + \frac{{\sigma_{{\mathrm{e}}}^{2} }}{{\overline{n}_{{\mathrm{R}}} }}}}\;{\mathrm{or}}\;h^{2} = \frac{{\sigma_{{\mathrm{g}}}^{2} }}{{\sigma_{{\mathrm{g}}}^{2} + \frac{{\sigma_{{\mathrm{e}}}^{2} }}{{\overline{n}_{{\mathrm{R}}} }}}},$$

where ${\sigma }_{\mathrm{g}}^{2}$ denotes the genotypic variance, ${\sigma }_{\mathrm{g}\times \mathrm{E}}^{2}$ denotes the interaction between genotype and environment, ${\sigma }_{\mathrm{e}}^{2}$ denotes the residual variance, $\overline{{n }_{\mathrm{R}}}$ denotes the average number of total replications per genotype from the whole experiment, and $\overline{{n }_{\mathrm{E}}}$ denotes the average number of environments in which the genotypes were evaluated.

All linear mixed models were fitted using ASReml-R package (version 4; Butler et al. 2023). The estimated BLUEs were then used in subsequent analyses. Pearson’s correlation coefficients between BLUEs and predicted performance were further analyzed using the R package corrplot (version 0.92; Wei and Simko 2017).

### Population structure analysis for the validation population

SNP markers were filtered separately for spring and winter populations using Plink (version 1.9; Purcell et al. 2007) with a minor allele frequency ≥ 0.05 and a missing rate < 0.10. Missing values were phased and imputed using Beagle (version 5.2; Browning et al. 2018). The final SNP count varied by the growth class following the aforementioned quality control process, where 113,242 and 133,936 SNPs were retained for spring and winter populations, respectively. For each tested SNP, homozygous for the most frequent allele, heterozygous, and homozygous for the alternative allele were coded as 0, 1, and 2, respectively. Principal component analysis (PCA) was implemented for all the genebank accessions by Plink, and the first two PCs were plotted against each other in order to portray the potential population structure due to the growth habit of the accessions. Rogers’ distance between genotypes among the core1000 collection, validation set, and their combined population was calculated by the R package poppr (Kamvar et al. 2013).

### Genome-wide association analysis (GWAS)

GWAS was conducted by integrating the genotypic data of the spring and winter validation sets with their corresponding phenotypic data, as well as with the expanded spring and winter population comprising the aforementioned core1000 collection and the validation set. To prevent over-correction of associations, both the Bayesian-information and linkage-disequilibrium iteratively nested keyway (BLINK; Huang et al. 2019) and linear mixed model (LMM) were used to identify significant SNPs for the three aforementioned diseases. The two models were implemented using Genome Association and Prediction Integrated Tool (GAPIT; Lipka et al. 2012) and Genome-wide Efficient Mixed-Model Association (GEMMA; Zhou and Stephens 2012), respectively. To control the population structure and reduce the inflation of false positive associations, the top three eigenvectors from the principal component analysis (PCA) and the kinship matrix were computed using the built-in pipelines embedded in GAPIT and GEMMA, respectively.

Significant associations threshold was defined using the Bonferroni correction (Holm 1979; 0.05/the number of SNPs) for each group to control false positives. Subsequently, Manhattan and combined quantile–quantile plots were generated using the R package CMplot (version 4.3.1; Yin et al. 2021). Thereafter, the proportion of the phenotypic variance (PVE) explained by a given SNP was estimated using a linear regression model, incorporating sample size, minor allele frequency, effect size, and the standard error of each SNP as described by Shim et al. (2015).

## Results

### Prediction abilities within the training set were evaluated by fivefold cross-validation

**Fig. 1:**
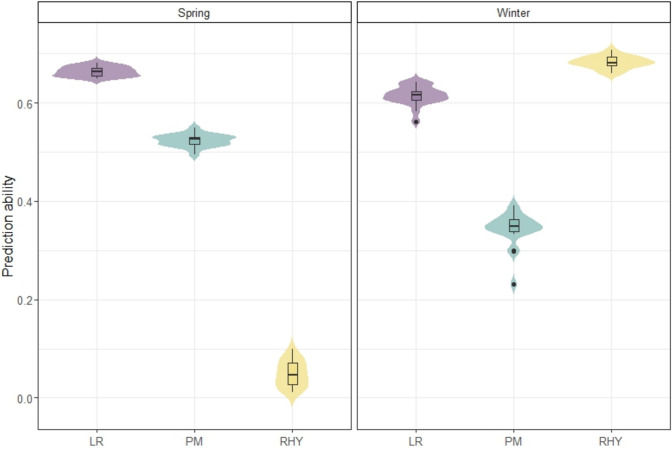
Fivefold cross-validation abilities of the genomic best linear unbiased prediction for *Blumeria graminis hordei* (PM), *Puccinia hordei* (LR), and *Rhynchosporium commune* (RHY), obtained in the core1000 collection, including 565 spring and 288 winter barley accessions

Genome-wide prediction models have been trained using published phenotypic (Yuan et al. 2024a) and genotypic (Milner et al. 2019) data of a barley genebank core1000 collection. To evaluate the prediction ability, we conducted fivefold cross-validation for spring and winter accessions separately. The prediction ability varied widely among the three disease resistance traits and the subpopulations defined by growth habit (Fig. 1). Within the spring panel, PM and LR showed a moderate prediction ability with an average of 0.52 and 0.66, respectively, whereas RHY failed to yield reliable predictions with an average prediction ability approaching zero, which likely reflects limited disease pressure, particularly in the spring populations, where dry conditions often prevent infection. Therefore, RHY in spring populations was not considered in the subsequent genome-wide prediction study. However, the pattern shifted in the winter panel, with RHY showing the highest prediction ability of on average 0.68. These contrasting patterns align with the trait-specific phenotypic variability and heritability present within each independent panel (Yuan et al. 2025). Consequently, these cross-validation results provide a useful benchmark for the expected performance of genome-wide prediction models, suggesting that the training set provides a reasonably reliable basis for predicting the disease resistance of non-phenotyped accessions within the IPK genebank.

### Genome-wide prediction of the performance for non-phenotyped genebank accessions and the selection of validation sets

**Fig. 2:**
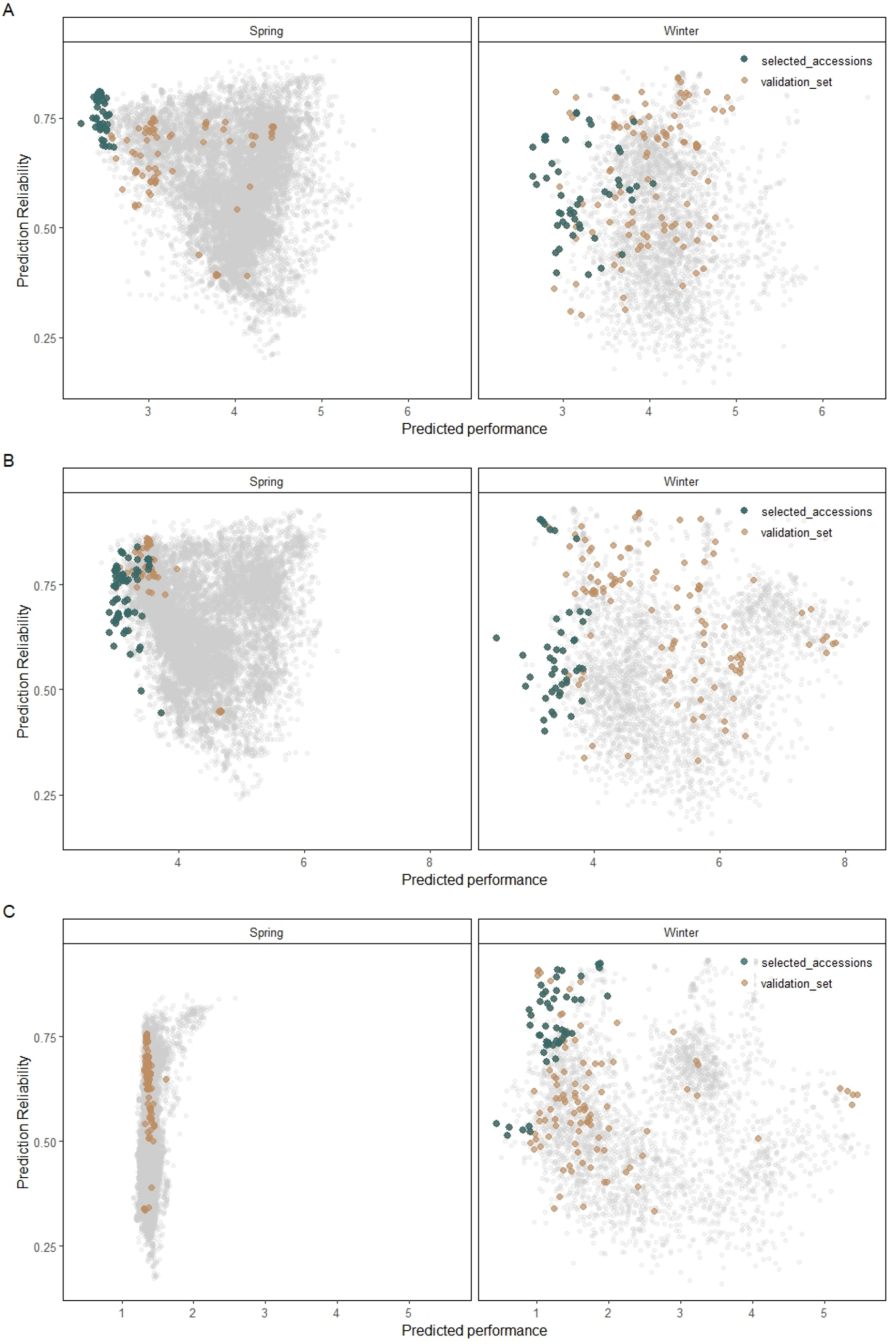
Predicted performance and prediction reliability for the resistance of *Blumeria graminis hordei* (**A**), *Puccinia hordei* (**B**), and *Rhynchosporium commune* (**C**) of spring and winter genebank accessions. The whole validation set was recolored as brown. Accessions selected based on the corresponding disease resistance traits were highlighted in dark green (color figure online)

Established prediction models trained on 565 spring and 288 winter barley accessions were used to estimate the performance of the non-phenotyped 16,560 spring and 3,680 winter barley genebank accessions, with their corresponding prediction reliabilities computed for each individual. The predicted performances exhibited a slightly higher mean value (4.14) for PM in the winter population, which was approximately 0.2 units higher than in spring. Overall, the two populations displayed nearly similar distribution profiles (Supplementary Fig. S2), with only a slight extension of the lower tail in spring, where approximately 4.78% of accessions fell below a resistance threshold of 3, compared to 0.82% in winter. LR and RHY both exhibited broader distributions of predicted performances in the winter population compared to spring. In the spring population, the predictions of LR were tightly clustered around the mid-range, resulting in a slightly smaller proportion of resistant lines (0.11%). In winter barley, the wider spread nonetheless corresponds to a similarly tiny fraction of resistant lines (0.15%). Overall, the reliability scores for all three traits in both populations span a wide range from 0.15 to 0.93 (Fig. 2), with associated standard errors from 0.10 to 1.14.

These aforementioned estimates informed the selection of 150 spring and 150 winter barley accessions from the IPK genebank, based on predicted phenotypic values less than 4 with reliability estimates larger than 0.5, with a few exceptions due to limited seed availability (Fig. 2; Supplementary Table S1). It is noteworthy that the validation set was selected based on a collective trait-specific selection strategy, whereby subpopulations were independently chosen based on their predicted resistance to each of the three targeted diseases. These subsets were then combined to form the final validation set. As a result, each selected genotype was predicted to be resistant to at least one of the target diseases, ensuring both trait relevance and efficient representation in the final set. For the winter barley validation set, 54, 46, and 50 genotypes were selected based on the predicted performance of PM, LR, and RHY resistance, respectively. In contrast, the combination of low heritability and poor cross-validated prediction ability for RHY within the spring training population precluded its use. Accordingly, 79 and 71 genotypes were strictly selected for PM and LR resistance, respectively.

### The validation set broadens the diversity space of the core collection

**Fig. 3:**
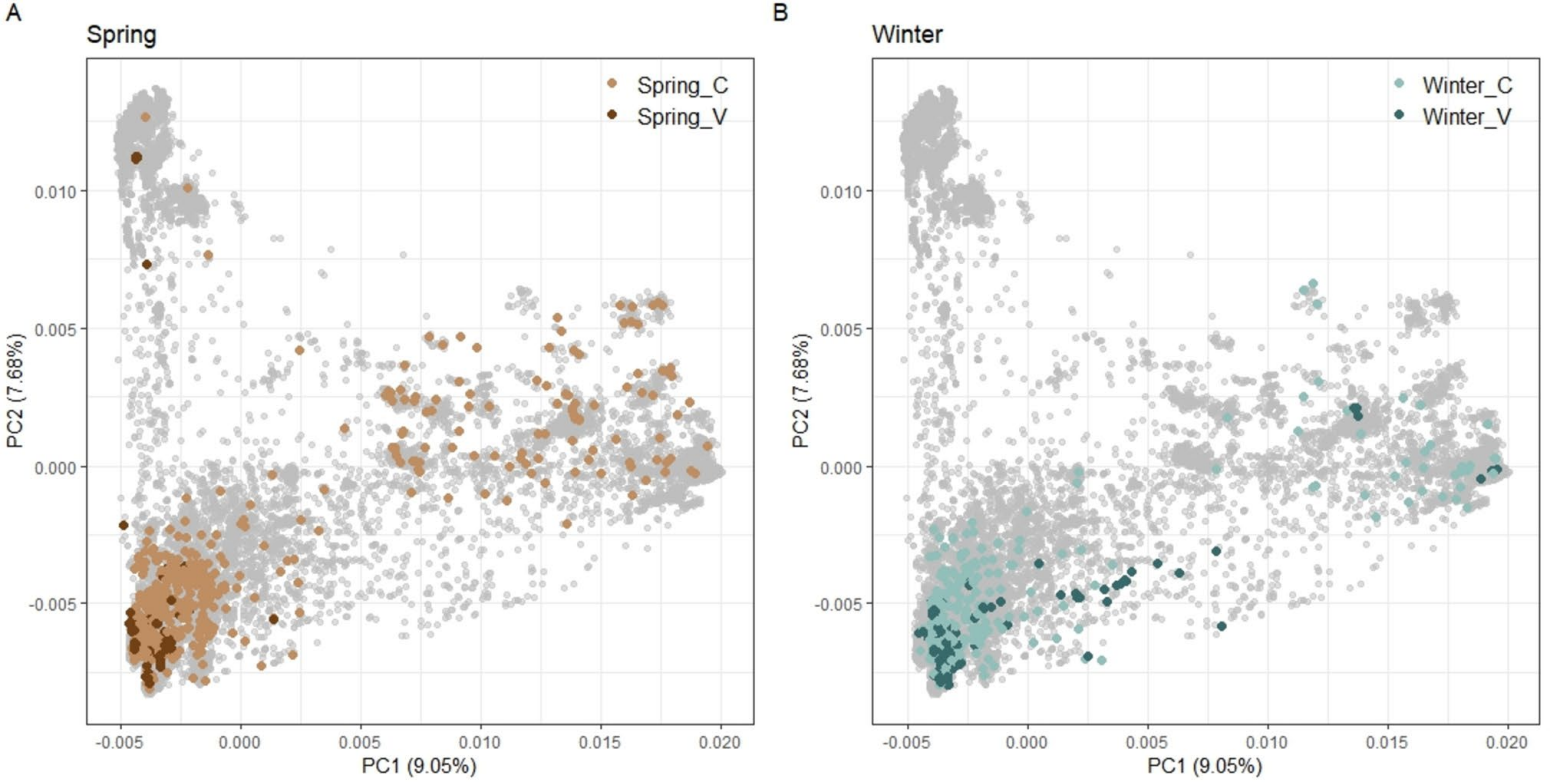
Genetic diversity analysis for the entire genebank barley collection including 20,458 genotypes. The principal component analysis (PCA) is portrayed in a bi-plot of the first two principal components for the whole collection. Spring_V, 150 spring barley of the validation set; Winter_V, 150 winter barley of the validation set; Spring_C, 565 spring barley of the core population; Winter_C, 288 winter barley of the core population

We investigated the genetic structure by applying principal component analysis (PCA) to the entire IPK genebank barley collection (Fig. 3). The first two PCs together explained 16.73% of the total genetic variation. Joint PCA of the aforementioned training and the validation set revealed that the spring population covered more diversity space than the winter subsets. This indicates greater genetic variation in the spring population and likely reflects its wider geographic distribution. However, no clear separation between distinct subpopulations was detected, except for a few divergent accessions. Those exceptions suggest subtle genetic differences between the two groups.

The validation set (150 spring and 150 winter) largely co-localizes with their respective core1000 collection, forming a single diffuse cluster and confirming the absence of discrete subpopulation structure. Notably, some genotypes, particularly within the spring subset, extended into regions not covered by the core1000 collection. This reflects our collective trait-customized selection strategy, in which subpopulations were independently selected based on predicted performance and reliability for each of the three target diseases, and subsequently combined. While this approach led to partial overlap with the genetic structure of the core1000 collection, it also contributed to an overall increase in genetic diversity by expanding the range of Rogers’ distance: For the spring population, incorporating the validation set into the core1000 collection slightly increased both the mean Rogers’ distance (from 0.481 to 0.492) and the standard deviation (from 0.103 to 0.124), indicating overall greater genetic dissimilarity with modestly increased variability in relatedness. In contrast, for the winter population, the mean Rogers’ distance decreased slightly (from 0.488 to 0.479) while the standard deviation increased (from 0.158 to 0.165), suggesting a broader spread of genetic relationships with both closer and more distant pairs introduced (Supplementary Fig. S3). Although the sampling strategy for the validation set was not designed to capture the full diversity of the broader collection, it deliberately introduced variation along dimensions relevant to disease resistance, thereby enhancing the representativeness and practical utility of the validation set for evaluation purposes.

### The observed phenotype of both validation sets showed high correlation with the predicted performance

To assess how well the genomic predictions translate to real-world performance, we conducted an experimental validation using the two sets of 150 spring and winter accessions, each evaluated across four locations during the 2023–2024 cropping season. The analysis of variance revealed highly significant genotypic effects (*p* ≤ 0.001) for traits with moderate-to-high heritability (Table 1). For the latter, environmental effects accounted for the largest proportion of the total variation, while genotypes and the genotype × environment interaction variances were comparatively smaller. Heritability estimates ranged from very low (*h*^2^ = 0.09) to high (*h*^2^ = 0.85) across traits. In particular, RHY in spring and PM in winter population showed the lowest heritabilities. This discrepancy is potentially attributed to several factors, including disparities in prevailing disease pressure and constraints imposed by the limited number of test sites, consistent with previous findings (McDonald et al. 1999; Linde et al. 2009; McDonald 2015). Taken together, the observed phenotypic variance and the moderate-to-high heritability estimates make the two validation sets well-suited for downstream genetic analyses.

**Table 1:** Analyses of variance for the studied traits in spring and winter validation set

Growth habit	Trait	*σ*^2^_g_	*σ*^2^_gxE_	*σ*^2^_E_	*N*_rep_	*h*^2^
Spring	PM	0.77^***^	1.09^***^	4.48^***^	4.99	0.71
	LR	0.30^**^	–	2.24^***^	2.99	0.36
	RHY	0.01^NS^	–	0.01^*^	1.99	0.09
Winter	PM	0.03^NS^	0.70^***^	3.75^***^	3.94	0.15
	LR	0.66^***^	0.50^***^	2.79^***^	4.97	0.75
	RHY	2.11^***^	0.97^***^	0.88^***^	4.95	0.85

*σ*^2^_g_: genotypic variance; *σ*^2^_gxE_: variance due to genotype-by-environment interaction; *σ*^2^_E_: variance due to environment; *N*_rep_: the average number of replications per genotype; *h*^2^: broad-sense heritability; PM: *Blumeria graminis hordei*; LR: *Puccinia hordei*; RHY: *Rhynchosporium commune*; ^**^ and ^***^: significance levels at *p* = 0.01 and 0.001, respectively; ^NS^: not significant

**Fig. 4:**
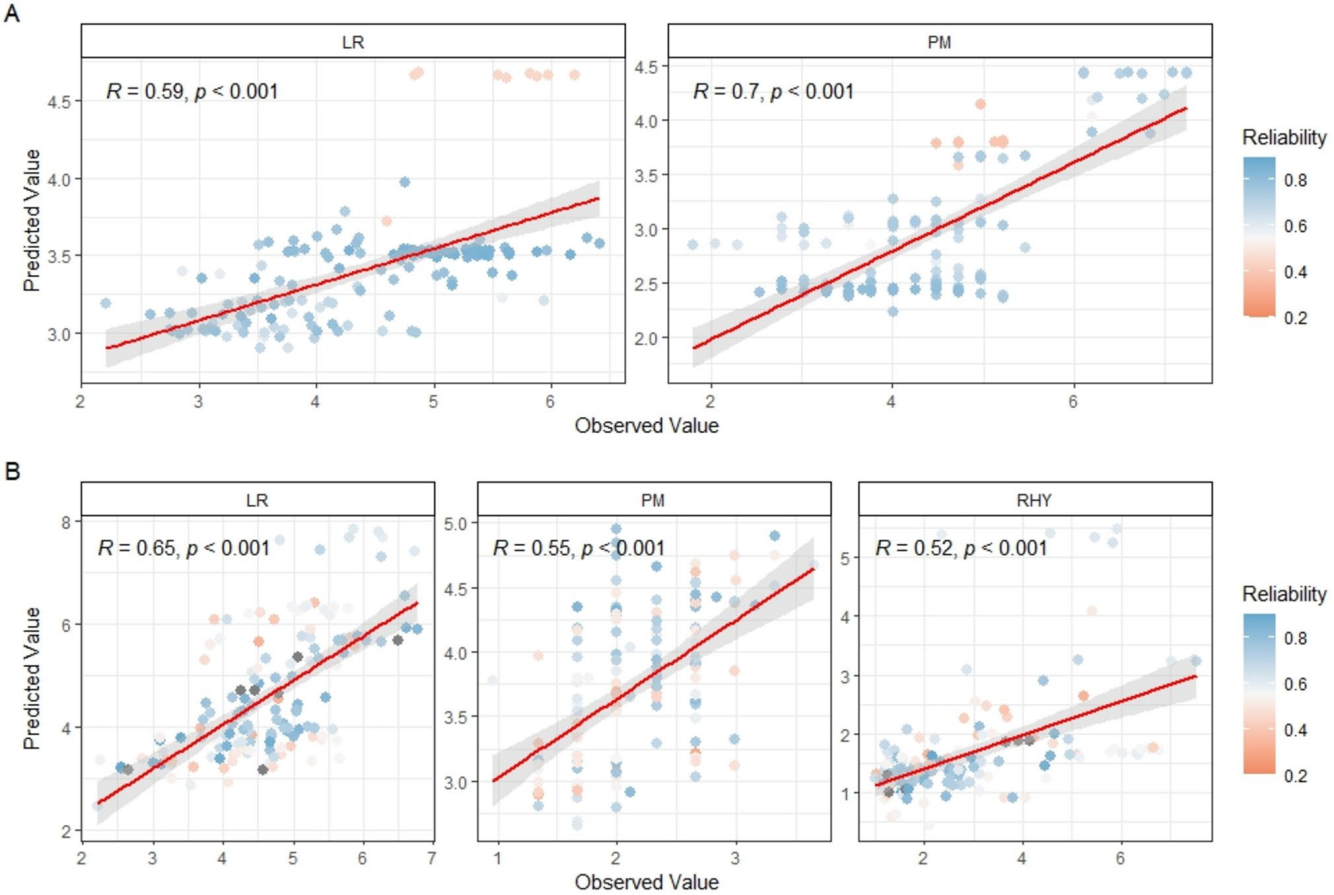
Pearson correlation coefficients (*r*) of observed and predicted phenotypic data in spring (**A**) and winter (**B**) validation set. PM: *Blumeria graminis hordei*; LR: *Puccinia hordei*; RHY: *Rhynchosporium commune*

Pearson’s correlation coefficient was computed to validate the observed and the predicted phenotype within the spring and winter populations, separately (Fig. 4). For PM and LR, moderate-to-strong correlations were observed in both spring (*r* = 0.70 and 0.59, *p* < 0.001) and winter (*r* = 0.55 and 0.65, *p* < 0.001) population, indicating consistent predictive performance across environments. For RHY in winter validation set, predicted and observed values aligned to a moderate degree (*r* = 0.52, *p* < 0.001). To assess the informativeness of the reliability estimates, correlations were also estimated using only genotypes with prediction reliability greater than 0.6. In this filtered subset, correlation values were generally higher (Supplementary Fig. S4). For instance, the correlation for LR in the spring population increased from 0.59 to 0.64, while that for PM remained the same. Similar improvements were observed in the winter population for both LR and RHY. These results underscore the practical value of incorporating reliability thresholds in genomic selection strategies, and that excluding low-reliability estimates can sharpen the predictive potential captured by the models. Overall, genomic selection based on prediction models can be effectively implemented to accelerate breeding progress by enabling early and accurate identification of superior genotypes.

### Genome-wide association analysis revealed QTLs associated with disease resistance traits in the validation set

Considering the pronounced diversity in population profiles (Fig. 3), GWAS analysis was performed separately for the two validation sets using GEMMA and BLINK, mitigating the risk of over-correction by the kinship matrix. The clustering of genotypes within each subgroup was controlled by considering the kinship matrix (*K*) and population structure (*Q*) as covariates. The observed and expected p values for the vast majority of markers matched, with a clear deviation of the observed values from the expected to the right end of the quantile–quantile plot, indicating that the *Q* + *K* model effectively controlled for false-positive associations (Supplementary Fig. S5).

A total of five marker–trait associations (MTAs) were detected for the three disease resistance traits in the validation set, spanning three chromosomes (Table 2; Supplementary Fig. S5). The individual significant SNPs explained 18.53 to 40.88% of the phenotypic variation, and the effect of each marker varied from − 1.17 to 0.93. Notably, three significant markers S1_518275616, S3_575138286, and S3_673991376 overlap with three high-confidence genes *HORVU1Hr1G077730*, *HORVU3Hr1G108010*, and *HORVU3Hr1G077890*, respectively. However, none of these MTAs were detected in the original training population, and only one (S3_575138286 associated with RHY resistance in the winter validation set) was detected when combining both validation set and training populations. Our results demonstrate that the potential of using an expanded, prediction-informed collection for association studies enriches resistant alleles not captured in the original training set, underscoring the dual value of genome-wide prediction for both accession selection and loci discovery.

**Table 2:** Significant markers identified in genome-wide association studies for three disease resistance traits in validation set

Growth habit	Trait	Model	SNP	Chr	Position (bp)	*p*_value	PVE (%)	Effect	MAF (*V*)	MAF (*V* + *T*)
Spring	PM	BLINK	S3_673991376	3	673,991,376	4.30E−08	40.25	0.32	0.26	0.41
Winter	LR	BLINK	S6_398388723	6	398,388,723	2.91E−10	32.83	0.58	0.14	0.28
		BLINK	S6_461896856	6	461,896,856	1.93E−08	18.23	−0.59	0.05	0.10
	RHY	BLINK	S1_518275616	1	518,275,616	1.82E−09	40.88	0.93	0.08	0.05
		BLINK/ GEMMA	S3_575138286	3	575,138,286	1.30E−08/ 1.19E−07	18.53	−0.82/ −1.17	0.13	0.14

PM: *Blumeria graminis hordei*; LR: *Puccinia hordei*; RHY: *Rhynchosporium commune*; PVE: phenotypic variation explanation; MAF (*V*): minor allele frequency in validation set; MAF (*V* + *T*): minor allele frequency in the expanded population, including validation set and training population

## Discussion

### Genome-wide prediction is a powerful tool to impute genebank information

Genebanks serve as reservoirs of untapped genetic diversity and offer the potential to improve climate resilience and stress adaptation traits (Leigh et al. 2022). However, large-scale phenotyping remains a major bottleneck. By harnessing genome-wide prediction to impute phenotypic data, we sidestep field evaluations and unlock the latent value of PGRs. Building on successful proof-of-concept studies in genebank predictions (Thorwarth et al. 2018; Jiang et al. 2021; Zhao et al. 2021), our work demonstrates the practical utility of this approach. Although phenotypic resistance data exist only for a fraction of the collection, GWP leverages the joint structure of the genomic and phenotypic information to infer resistance values across the entire collection, thereby overcoming the phenotyping bottleneck and enabling collection-wide trait characterization.

Although various GWP frameworks exist, their performance within-genebank setting is largely comparable, with GBLUP showing a stable performance (El Hanafi et al. 2023). We therefore used the latter as the default model to impute phenotypic values for all non-phenotyped spring and winter barley accessions at IPK genebank. The mean prediction reliability for the non-phenotyped accessions was high (> 0.5) for all traits considered (Supplementary Fig. S2B), suggesting that the predictions for these accessions are reasonably informative. By selecting a 300-genotype validation set based on these reliabilities and predicted disease score, we then demonstrated acceptable concordance between the observed and predicted performance across the four contrasting environments (Fig. 4). Together, our findings highlight the potential of GWP as a fast, cost- and time-effective proxy for large-scale phenotyping, which can streamline pre-breeding workflows and accelerate the targeted introgression of novel disease resistance alleles into elite germplasm.

Despite the promising results obtained by GBLUP, exploring strategies to further enhance prediction accuracy remains important. For instance, incorporating population structure or geographic origin as covariates has been shown to improve model performance in structured populations (Neyhart et al. 2022). Additionally, integrating functional trait-specific markers can help target loci with known biological effects (Spindel et al. 2015). Diagnostically informative loci, which confer recessively inherited broad-spectrum resistance like *Mlo* for powdery mildew resistance (Kusch and Panstruga 2017), *RPH1* to *RPH19* for leaf rust resistance (Park et al. 2015; Martin et al. 2020), and *Rrs15* to *Rrs17* for *Rhynchosporium commune* resistance (Zhang et al. 2020)*,* could be explicitly weighted in prediction models. This hybrid approach, bridging marker-assisted and genomic selection, yielded significant improvements for traits like heading date and plant height in hybrid wheat (Zhao et al. 2014), and slightly enhanced accuracy in a hybrid maize when including MTAs as cofactors (Li et al. 2020). However, our current genotyping-by-sequencing data lack the density to capture these causal variants at key loci; therefore, advances in high-density SNPs and whole-genome sequencing, together with integrating InDels and expanding catalogs of validated functional genes, may enable its trait-specific accuracy gains across genebank collections.

### Core collection as an effective training set for accurate within-genebank genome-wide prediction

For genome-wide prediction, constructing an effective training set is crucial and hinges on striking the right balance between genetic diversity, trait architecture, and practical constraints such as limited phenotyping capacity (Isidro et al. 2015), yet empirical studies have shown that core collections may remain effective under such conditions. Leveraging the genomic relationship matrix, core collections were designed to maximize genetic variance and predictive power while minimizing expected prediction error (Akdemir and Isidro-Sánchez 2019). This was also the case for the IPK genebank, where core collections were generated to represent the molecular diversity of the entire genebank collection for barley (Milner et al. 2019), wheat (Schulthess et al. 2022), and garlic (Colmsee et al. 2012). In the present study, the barley core1000 collection demonstrated promising cross-validated prediction performance (Fig. 1), highlighting the practical feasibility of genomic prediction from such subsets.

Although core collections represent only a fraction of the entire genebank, their optimized composition enables efficient prediction. Remarkably, Jiang et al., (2021) showed that using a core collection representing only 1/15 of its original training population size resulted in only a moderate decrease in prediction accuracy, illustrating that smaller, optimized training sets can remain efficient. This mirrors the calibration-set optimization approach, where carefully chosen reference individuals can replace larger, more expensive panels (Rincent et al. 2012). However, the extent to which prediction accuracy can be maintained with smaller training sets depends critically on the trait under consideration and the population’s diversity, as genebank materials typically exhibit a much broader range of phenotypic values than commercial populations (Lell et al. 2025). Crossa et al. (2016) further demonstrated that phenotyping a limited subset (20% of the collection) while genotyping the entire population can yield relatively high prediction accuracies for traits with moderate-to-high heritability, thereby validating the genomic selection approach for genebank accessions.

However, the core1000 collection used in our study was selected solely based on the genotypic data (Milner et al. 2019), so the resulting phenotypic distribution for three disease traits was skewed toward susceptibility. A training set that underrepresents favorable or extreme phenotypes risks introducing systematic bias into the model, thereby constraining prediction accuracy and reducing its generalizability to the broader genebank. This concern is consistent with the findings of Akdemir and Isidro-Sánchez (2019), who showed that a training population selected without reference to the test set can perform suboptimally, whereas targeted designs that consider phenotypic representativeness significantly improve predictive ability. This underscores the need for dynamic strategies that iteratively expand core collections as new phenotypic data become available, ensuring prediction models remain robust as genebank collections expand.

### Strategic validation set selection for multiple traits enables the discovery of major-effect loci

Genome-wide prediction enables direct access to the untapped diversity in genebanks (Crossa et al. 2016; Jiang et al. 2021), facilitating the identification of accessions with favorable performance and high prediction reliability for pre-breeding (Gorjanc et al. 2016). However, the tendency to prioritize genotypes with high prediction reliability can inadvertently narrow the genetic base by excluding diverse or underrepresented accessions. This trade-off highlights the need for selection strategies that balance predictive accuracy with genetic breadth. Incorporating multiple traits in the selection process expands breeding relevance by capturing a broader spectrum of beneficial alleles (Berkner et al. 2024). To address the distinct genetic architectures of the three disease resistance traits and maximize discovery power, we implemented a collective trait-customized selection strategy. This ensured that each selected genotype was predicted to be resistant to at least one target disease, resulting in a panel that was both trait-relevant and resource-efficient. By prioritizing genotypes with complementary resistance profiles, the validation set captured more neutral genetic diversity (Supplementary Fig. S3); variation not directly linked to the target traits but crucial for preserving rare alleles, maintaining long-term adaptability, and enhancing the power of GWAS. It also introduced genotypes with more extreme phenotypic performance across multiple traits (Supplementary Table S2). This dual benefit highlights the strength of multi-trait selection in delivering both immediate gains in prediction accuracy and long-term genetic stewardship, positioning genebank collections as engines of discovery, GWAS-enabled insights, and sustainable breeding.

GWAS statistical power generally increases with population size, heritability, and allele frequency (Myles et al. 2009). Simulations and empirical benchmarks indicated that a range of 100–500 individuals is typically sufficient to perform GWAS (Korte and Farlow 2013; Gawenda et al. 2015; Alqudah et al. 2020). In particular, even modestly sized populations can reliably detect major-effect QTL when those explain a high fraction of phenotypic variance (> 10%). For instance, a flowering time QTL associated with variation in the vernalization response gene *FRIGIDA* was identified in a population of just 107 *Arabidopsis thaliana* genotypes (Atwell et al. 2010). Similarly, in our study, five MTAs were detected, each explaining more than 18% of the phenotypic variation. Three of these SNPs overlapped with high-confidence genes. In particular, the marker S1_518275616, associated with RHY resistance in the winter population, falls within the coding region of *HORVU1Hr1G077730*, a FRS/FRF-type transcription factor implicated in plant immunity for both biotic and abiotic stresses (Jafari and Dolatabadian 2025). These results demonstrate that, when favorable conditions such as sufficiently frequent and impactful alleles are present, even moderately sized validation sets can yield robust and informative GWAS outcomes, providing clear targets for downstream genetic and functional analysis. From a breeding perspective, they represent important resources for broadening the resistance base in elite germplasm, particularly as many of these rare alleles originate from underutilized genebank accessions.

### Context dependence of marker–trait association in diverse populations

GWAS was conducted separately across three population sets: the validation set, the training population, and their combination, for both spring and winter barley. Notably, five marker–trait associations (MTAs) were detected exclusively in the validation sets and not in the training population (Supplementary Table S3), despite the resistance-associated alleles being segregating in both. This indicates that prediction-guided sampling can enrich for genotypes carrying informative variants that were underrepresented in the core collection, thereby enhancing discovery power. Furthermore, among the five MTAs, only one (S3_575138286 for RHY resistance in the winter validation set) was also detected in the combined population (Supplementary Table S3), despite modest increases in minor allele frequencies (Table 2). This discrepancy likely reflects dilution of effect sizes as minor allele frequencies rise, compounded by greater phenotypic variation, more complex population structure, and environmental heterogeneity, thus weakening the strength of association in larger populations. Supporting this, a large-scale GWAS on type 2 diabetes found that variants with lower MAFs tend to have larger effect sizes (Mahajan et al. 2018).

Such context dependence, where effect magnitude and detectability vary with population makeup and trial conditions, highlights the need to validate MTAs across diverse panels and environments before deploying them in breeding programs. Extremely rare alleles with moderate effects often evade detection in standard GWAS (Myles et al. 2009), while common variants already fixed in modern varieties have largely exhausted their contribution to trait improvement, limiting their relevance for identifying new donor sources. To capture the full allelic spectrum, the future validation set could be expanded based on a broader range of prediction reliability and performance, to improve the chances of uncovering new and informative genetic variation. Additionally, pathogen populations may shift over time and complicate the detection and replication of associations across years; this potential source of variability could not be evaluated in our study due to the one-year data limitation. This temporal variability highlights the need for repeated multi-year trials and dynamic validation strategies that account for evolving biotic pressures for broad adaptation as a breeding target.

### From prediction to practice: leveraging validation set for core collection expansion

In practice, breeders must balance the risk of advancing untested lines against the cost of large-scale trials. The validation set provides a natural buffer of untested material whose predicted performance and associated reliability can be quantified before committing to expensive phenotyping. Yet, beyond this conventional role, our results show that the validation set can, and should, play an active, iterative part in expanding the genetic diversity as well as the phenotypic representation of the core collection (Supplementary Fig. S3; Supplementary Table S2) and refining prediction models through an iterative feedback process.

Our comparison of three training set configurations revealed a nuanced balance between exploiting high reliability estimates and anchoring models in real observations (Supplementary Fig. S6). Augmenting the training set with predicted values from the validation set led to up to a 1.5-fold improvement in prediction reliability, with the effect most pronounced in the winter population (*p* < 0.0001; Supplementary Fig. S6A), suggesting that, when true phenotypes are unavailable, high-reliability imputed data can meaningfully bootstrap model accuracy. However, in the spring population, introducing even a modest number of observed validation phenotypes outperformed purely predicted augmentation (with the exception of LR), indicating that limited ground-truth data can more effectively anchor the model and reduce bias in prediction. This divergence underscores the central trade-off in genomic predictions between breadth (many imputed values) and depth (few true observations). Our data show that breadth-focused augmentation sharpens model discrimination, as the wider dispersion of predicted resistance values reflects finer resolution among accessions (Supplementary Fig. S6B), whereas depth-focused addition stabilizes predictions, especially in genetically diverse panels. In practice, the optimal balance may depend on population structure: Observed data contributed less to the winter panel due to its narrower genetic base, whereas the more diverse spring panel benefited substantially. Taken together, these findings highlight the dual value of the validation set: as a tool for targeted diversity expansion and as a dynamic lever for model improvement. From a pre-breeding perspective, integrating prediction-informed sampling into training set design supports the development of core collections that go beyond neutral representativeness to include functionally enriched, trait-relevant diversity. This allows genebank resources to be leveraged more effectively through balancing statistical power, phenotyping efficiency, and genetic representativeness to support both immediate and long-term breeding goals. We therefore advocate for an iterative framework that continuously integrates predicted and observed data to optimize selection, discovery, and diversity management (Belamkar et al. 2018).

Nevertheless, several caveats warrant discussion. First, the effectiveness of imputed augmentation depends on the reliability of predictions, as overconfident estimates may mislead candidate selection. Second, the genetic architecture of target traits (e.g., heritability, genotype × environment interaction sensitivity) will influence the relative benefits of each approach. For traits with strong genotype-by-environment interactions, multi-trait or environment-informed validation schemes may better capture relevant variation. Third, population structure must be explicitly accounted for. While our stratified sampling by spring versus winter types ensured equitable performance estimates, more complex pedigrees or subpopulations (e.g., row type, geographic origin) may require customized stratification strategies. Beyond statistical considerations, factors such as rare alleles with large effects, copy number variations, or chromosomal rearrangements can also contribute to prediction inaccuracies. Identifying and investigating outlier accessions with discrepancies between predicted and observed values provides an opportunity to uncover novel biological mechanisms underlying trait variation (De Los Campos et al. 2013), ultimately improving model accuracy and enhancing our understanding of complex trait architecture.

## Supplementary Information

Below is the link to the electronic supplementary material.

Supplementary file1 (DOCX 10668 KB)

Supplementary file2 (XLSX 41 KB)

## Data Availability

The raw phenotypic data described here as well as the ready-to-use phenotypic values (BLUEs) for spring and winter validation set, and the R script to import and curate the raw phenotypic data to compute heritability and BLUEs are available in the e!DAL-PGP Repository (Arend et al. 2016) and can be directly accessed here (Yuan 2025).
