# Supplementary material for: Targeted expansion of a barley genebank core collection facilitates the discovery of disease resistance loci
Source: Theor Appl Genet. 2026 Jan 11;139(1):30. doi: 10.1007/s00122-025-05139-9 (PMC12791075; doi:10.1007/s00122-025-05139-9)
Supplement: Supplementary file 1 — Supplementary file1 (DOCX 10668 KB) [file 122_2025_5139_MOESM1_ESM.docx]

**
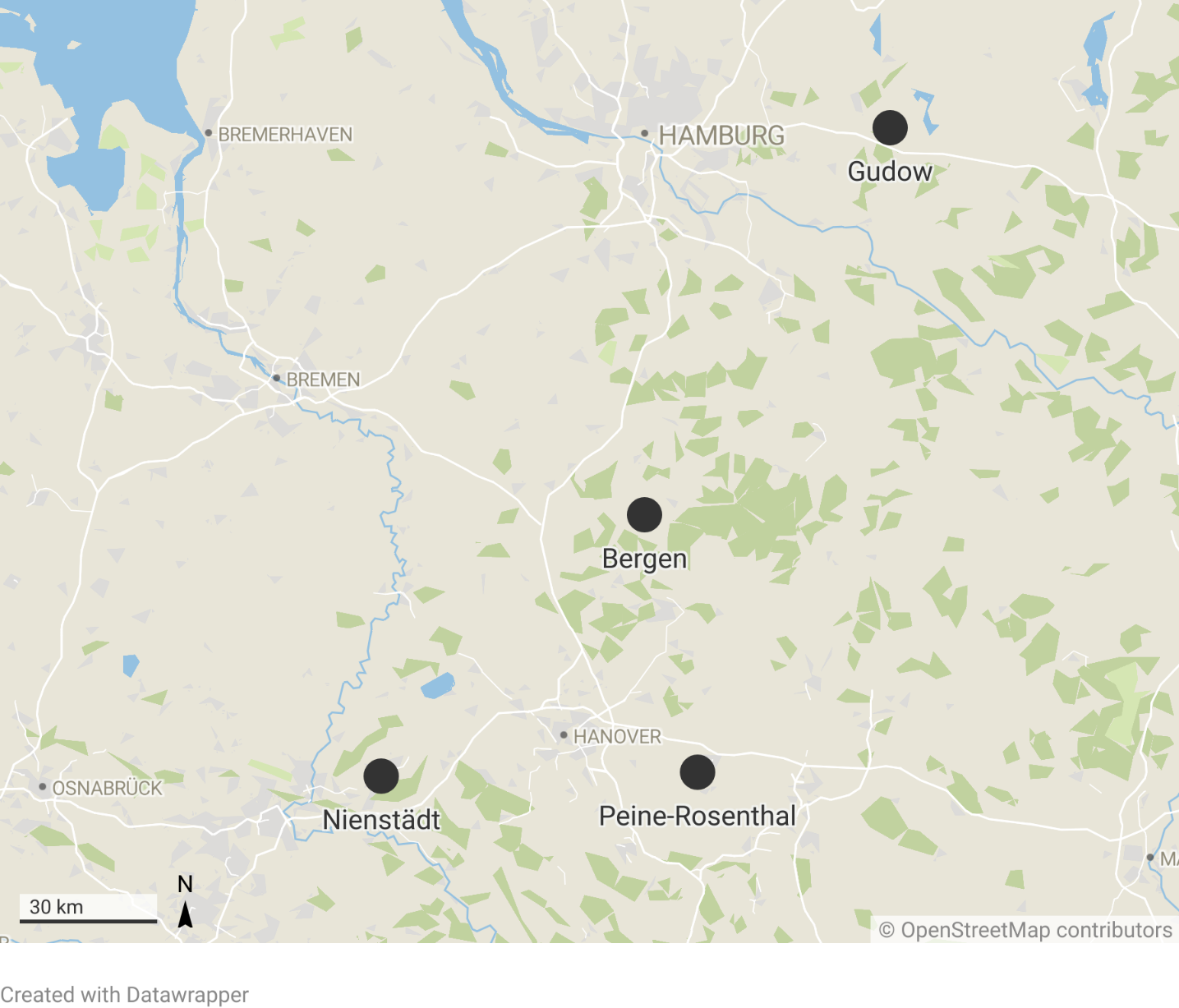
**

**Supplementary Fig. S1** Geographic locations of the four validation sites where the experiments were conducted.

**
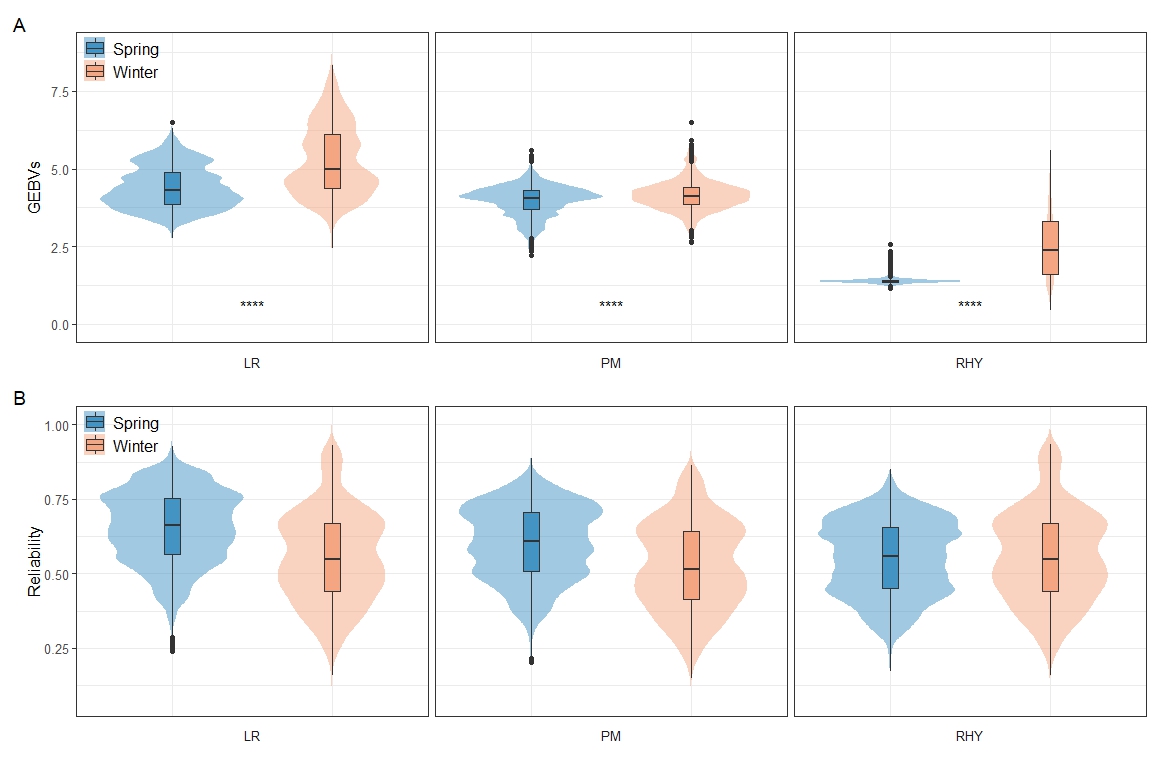
Supplementary Fig. S2** Genomic estimated breeding values (A) and Prediction reliability (B) for three disease resistance traits of non-phenotyped spring and winter population. GEBVs: genomic estimated breeding values; PM: *Blumeria graminis hordei*; LR: *Puccinia hordei*; RHY: *Rhynchosporium commune*; ^****^: significance levels at *p* = 0.0001.

**
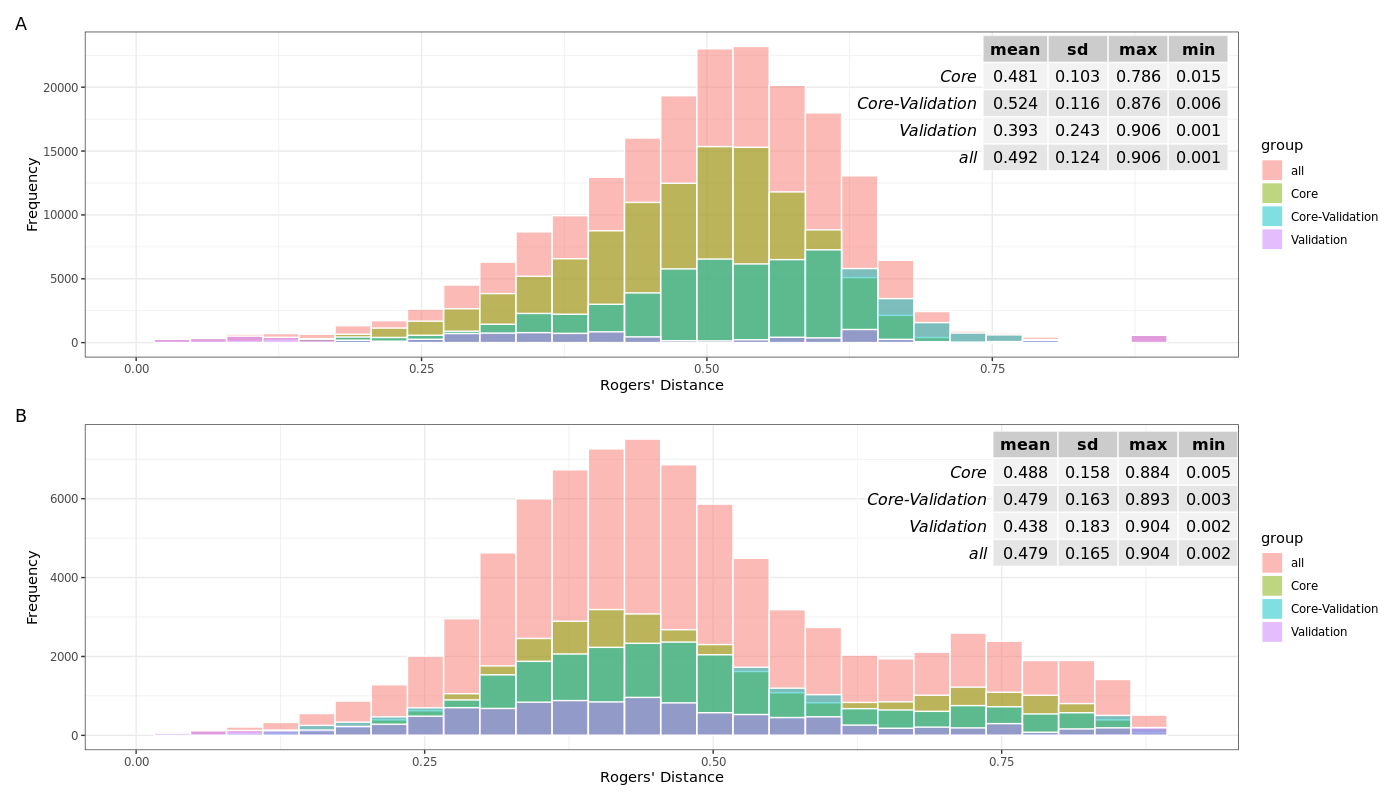
Supplementary Fig. S3** Distribution of Rogers’ distance between genotypes in (A) the spring population and (B) the winter population. Bars show the frequency distribution for all pairwise comparisons (pink) and for specific subsets: core collection (yellow-green), core collection *vs.* validation set (blue-green), and validation set (purple). The inset tables summarize the mean, standard deviation (sd), maximum (max), and minimum (min) distances for each group.

**
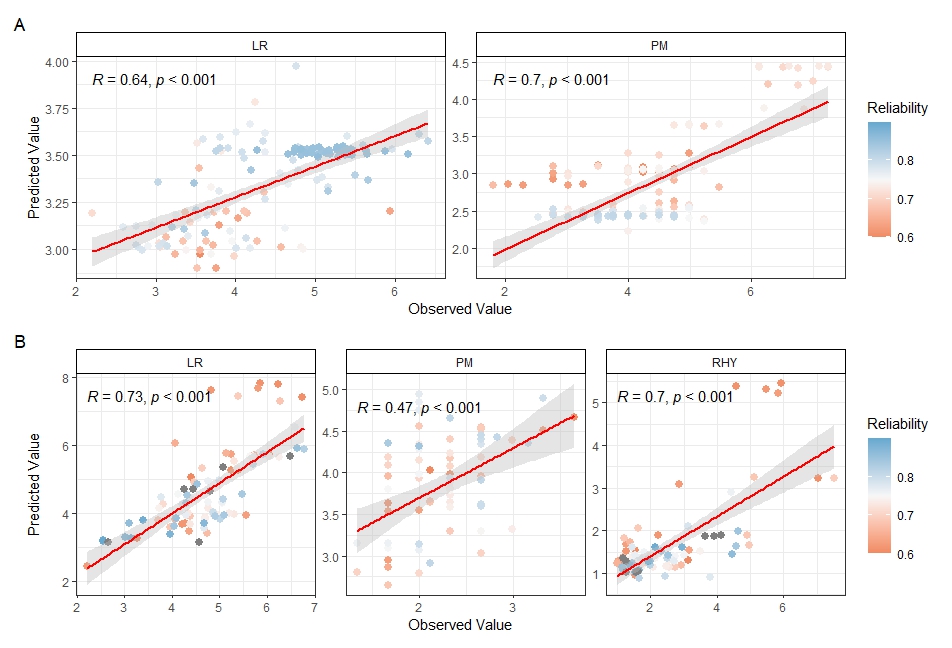
Supplementary Fig. S4** Pearson correlation coefficients (r) of observed and predicted phenotypic data in spring (A) and winter (B) validation set with prediction reliability larger than 0.6. PM: *Blumeria graminis hordei*; LR: *Puccinia hordei*; RHY: *Rhynchosporium commune*.

**Supplementary Fig. S5** Quantile–quantile plot (left side) and Manhattan plots (right side) of the genome association analysis results in validation set of the resistance of *Blumeria graminis hordei* in spring population by BLINK (A), *Puccinia hordei* in winter population by BLINK (B), and *Rhynchosporium commune* in winter population by GEMMA and BLINK (C). The black horizontal dashed line in Manhattan plots corresponds to the significance threshold.


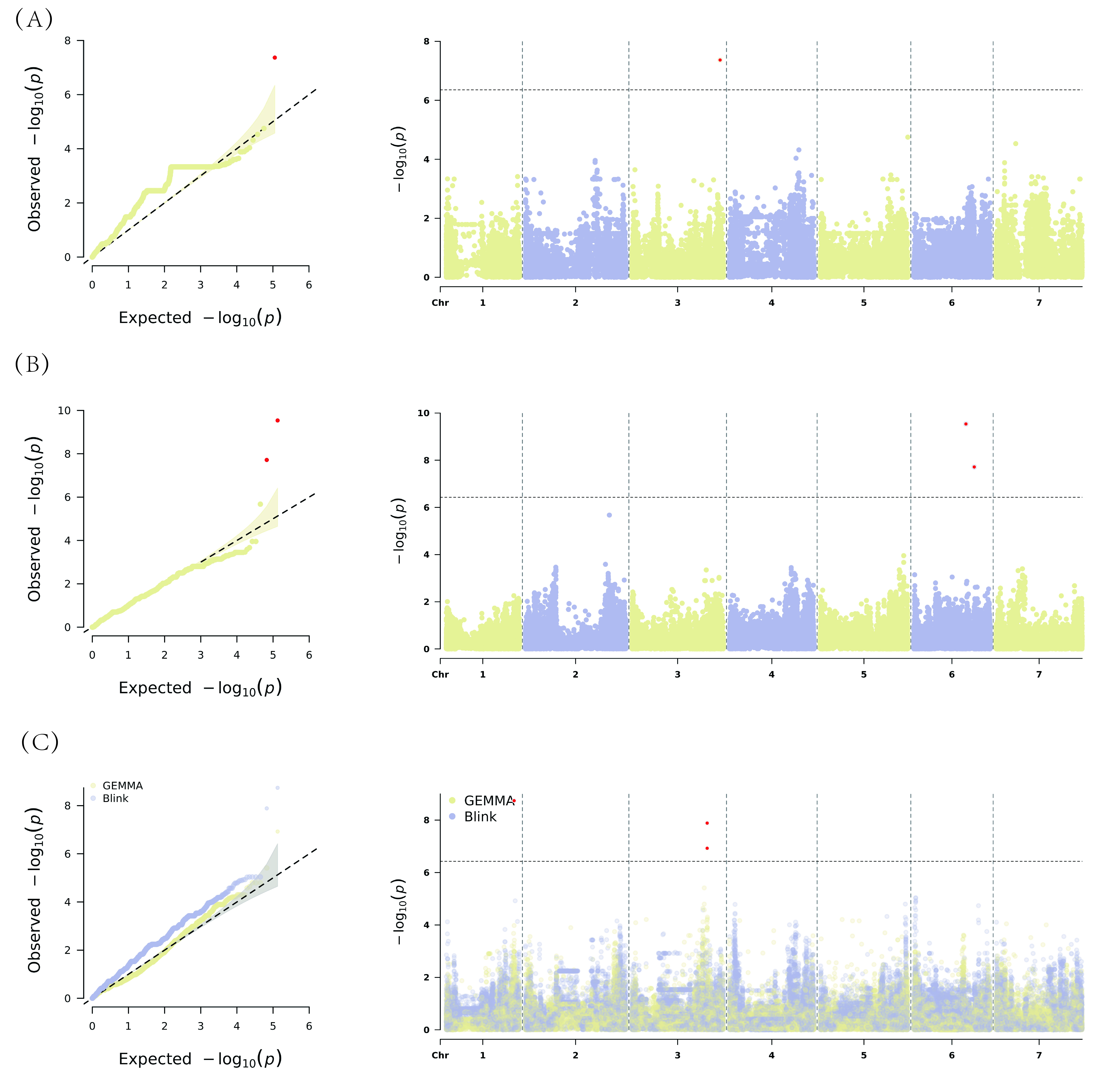


**
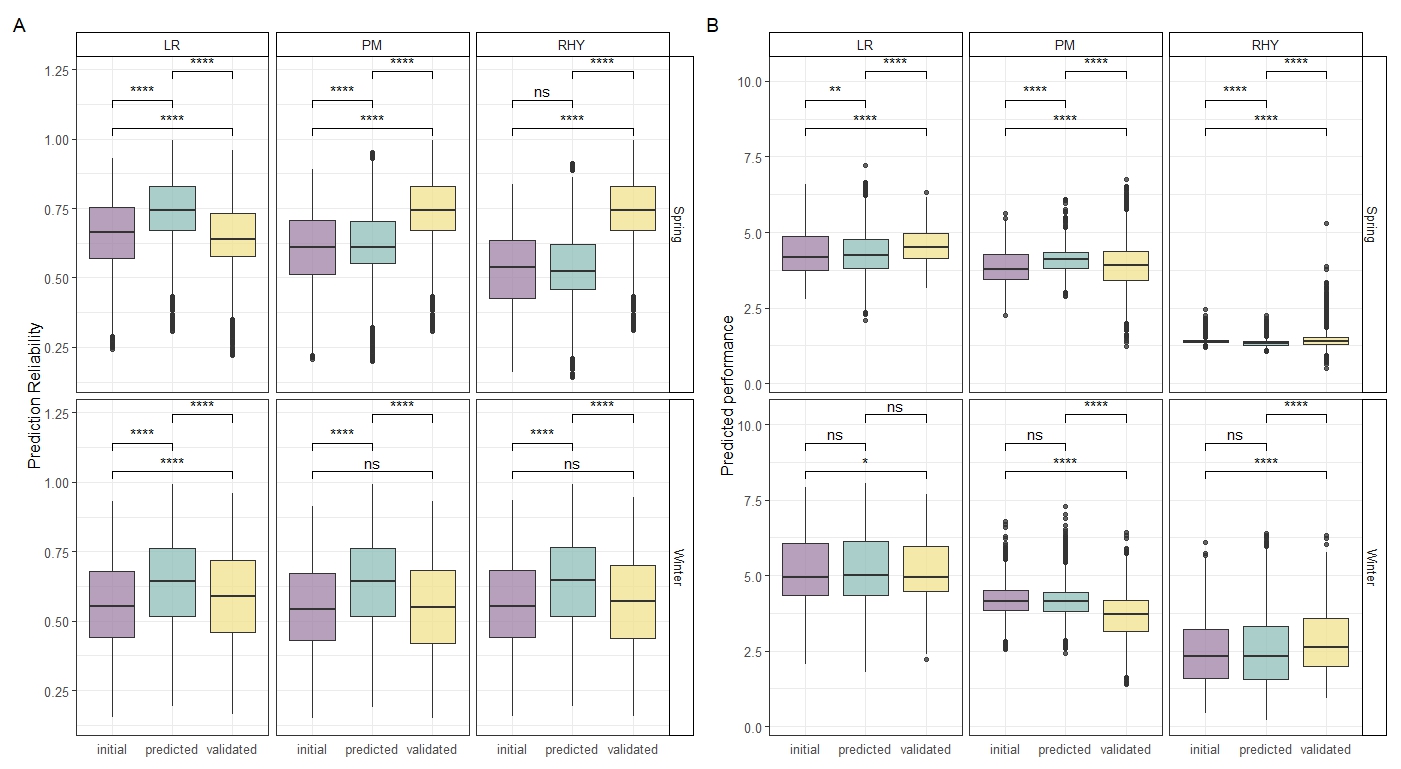
Supplementary Fig. S6** Prediction reliability (A) and predicted performance (B) for three disease resistance traits of non-phenotyped spring and winter population using initial core collection as training set (initial); augment training set adding predicted performance of validation set (predicted); augment training set adding the observed value of validation set (validated). PM: *Blumeria graminis hordei*; LR: *Puccinia hordei*; RHY: *Rhynchosporium commune*; ^****^: significance levels at *p* = 0.0001; ^ns^: not significant
